# Structure and Reactivity of *Bacillus subtilis* MenD Catalyzing the First Committed Step in Menaquinone Biosynthesis

**DOI:** 10.1016/j.jmb.2010.06.025

**Published:** 2010-08-13

**Authors:** Alice Dawson, Minjiao Chen, Paul K. Fyfe, Zhihong Guo, William N. Hunter

**Affiliations:** 1 Division of Biological Chemistry and Drug Discovery, College of Life Sciences, University of Dundee, Dow Street, Dundee DD1 5EH, UK; 2 Department of Chemistry, Center for Cancer Research, The Hong Kong University of Science and Technology, Clear Water Bay, Kowloon, Hong Kong SAR, China

**Keywords:** CoA, coenzyme A, PDB, Protein Data Bank, SAD, single-wavelength anomalous diffraction, SEPHCHC, 2-succinyl-5-enolpyruvyl-6-hydroxy-3-cyclohexadiene-1-carboxylate, SeMet, selenomethionine, ThDP, thiamine diphosphate, PEG, polyethylene glycol, crystal structure, enzyme mechanism, menaquinone biosynthesis, thiamine diphosphate cofactor

## Abstract

The first committed step in the classical biosynthetic route to menaquinone (vitamin K_2_) is a Stetter-like conjugate addition of α-ketoglutarate with isochorismate. This reaction is catalyzed by the thiamine diphosphate and metal-ion-dependent 2-succinyl-5-enolpyruvyl-6-hydroxy-3-cyclohexadiene-1-carboxylate synthase (MenD). The medium-resolution (2.35 Å) crystal structure of *Bacillus subtilis* MenD with cofactor and Mn^2+^ has been determined. Based on structure–sequence comparisons and modeling, a two-stage mechanism that is primarily driven by the chemical properties of the cofactor is proposed. Hypotheses for the molecular determinants of substrate recognition were formulated. Five basic residues (Arg32, Arg106, Arg409, Arg428, and Lys299) are postulated to interact with carboxylate and hydroxyl groups to align substrates for catalysis in combination with a cluster of non-polar residues (Ile489, Phe490, and Leu493) on one side of the active site. The powerful combination of site-directed mutagenesis, where each of the eight residues is replaced by alanine, and steady-state kinetic measurements has been exploited to address these hypotheses. Arg409 plays a significant role in binding both substrates while Arg428 contributes mainly to binding of α-ketoglutarate. Arg32 and in particular Arg106 are critical for recognition of isochorismate. Mutagenesis of Phe490 and Ile489 has the most profound influence on catalytic efficiency, indicating that these two residues are important for binding of isochorismate and for stabilizing the cofactor position. These data allow for a detailed description of the structure–reactivity relationship that governs MenD function and refinement of the model for the catalytic intermediate that supports the Stetter-like conjugate addition.

## Introduction

Menaquinone, or vitamin K_2_, is a lipid-soluble molecule with distinct functions according to the type of organism in which it is found. Its chemical properties are exploited in the electron transport (respiratory) chain in Gram-positive aerobes and facultative Gram-negative bacteria under anaerobic conditions.^1^ Gram-negative bacteria use menaquinone only under anaerobic conditions, using ubiquinone under aerobic conditions.^2^ In mammals, which acquire menaquinone from intestinal microflora and diet, it is an important cofactor for glutamate γ-carboxylation, an essential posttranslational modification of proteins involved in blood coagulation, bone metabolism, and vascular biology.^3^

Menaquinone biosynthesis has been most studied in *Escherichia coli*.^1^ The starting point is chorismate, the branch point intermediate of the Shikimate pathway.^4^ Chorismate is first isomerized to isochorismate and then converted, in three stages, to *o*-succinyl-1-benzoate. Two further enzyme-catalyzed reactions, involving ATP and coenzyme A (CoA), lead to a CoA-naphthalene derivative before a recently identified thioesterase removes the CoA moiety to release 1,4-dihydroxy-2-napthanoate.^5^ Prenylation, followed by methylation, then produces menaquinone. An alternative and distinct route to menaquinone exists in a small subset of bacteria such as *Helicobacter pylori*.^6^ The starting point remains chorismate but the intermediates include an inosine derivative, futalosine.

**Fig. 1:**
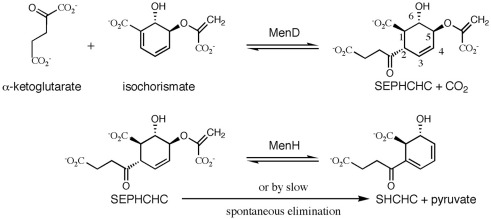
The reactions catalyzed by MenD and MenH in menaquinone biosynthesis.

Our interest centers on 2-succinyl-5-enolpyruvyl-6-hydroxy-3-cyclohexadiene-1-carboxylate (SEPHCHC) synthase (EC 2.2.1.9) called MenD, the enzyme that catalyzes the first committed step of the classical menaquinone biosynthetic route (Fig. 1). Initially, the thiamine diphosphate (ThDP)-dependent MenD was misassigned as a bifunctional 2-succinyl-6-hydroxy-2,4-cyclohexadiene-1-carboxylate synthase^7^ catalyzing decarboxylation of α-ketoglutarate and then ligation of a second substrate, isochorismate, to produce SEPHCHC. Cleavage of the pyruvate moiety then produced 2-succinyl-6-hydroxy-2,4-cyclohexadiene-1-carboxylate. Subsequent studies revealed that MenD is a monofunctional SEPHCHC synthase and the only enzyme known to catalyze addition of a ThDP intermediate to the β-carbon of a second substrate, in a Stetter-like reaction, which is a 1,4-conjugate addition of an aldehyde to a β-unsaturated compound.^8^ A complication in previous studies was the spontaneous release of pyruvate from SEPHCHC giving the misleading perception that a bifunctional enzyme was present.^9,10^ Jiang *et al.* showed that the enzyme encoded by the *menH* gene is actually responsible for the catalytic cleavage of pyruvate from SEPHCHC (Fig. 1).^9^

The majority of genes that code for enzymes involved in menaquinone biosynthesis, including *menD*, have been shown by gene knockout to be essential in *Bacillus subtilis*.^11^ Such methods also indicate essentiality of MenD in *Haemophilus influenzae*^12^ and *Mycobacterium tuberculosis*.^13^ Since these are two important pathogens, then the understanding of MenD function and activity has potential to inform on early-stage drug discovery research.^14^

The structure of MenD from *E. coli* in complex with its cofactor ThDP,^15^ the apo structure of *Ec*MenD,^16^ and a complex with oxoglutaric acid in the absence of cofactor^17^ have been determined. In the latter structure, the ligand binds at the site normally occupied by the ThDP diphosphate and so does not provide any information about substrate binding. We now report the medium-resolution crystal structure of the *B. subtilis* enzyme (*Bs*MenD), solved by targeting the anomalous dispersion signal from a selenomethionine (SeMet) derivative. A comparison of structures and sequences of MenD orthologues guided molecular modeling, which has allowed us to generate hypotheses concerning the functional roles for eight residues in the active site. A detailed kinetic analysis of the wild-type enzyme and eight point mutations serve to address questions concerning recognition and processing of substrate and have allowed us to elucidate the structure–reactivity relationship that governs MenD activity.

## Results and Discussion

### General comments

*Bs*MenD crystallizes in an orthorhombic system, space group *P*2_1_2_1_2_1_, with four polypeptide chains, labeled A to D in the asymmetric unit. The mass of protein in the tetramer is about 256.4 kDa. The structure was solved by use of single-wavelength anomalous diffraction (SAD) methods and refined to 2.35 Å resolution. The crystallographic statistics and model geometry (Table 1) indicate that the analysis has produced an acceptable medium-resolution model. Continuous and well-defined electron density was observed for each polypeptide chain from Thr2 to Leu580, except for chain C where the final two residues were not observed. Overlay of the chains using Secondary-Structure Matching^20^ gives root-mean-square deviations (r.m.s.d.'s) in the range 0.22–0.30 Å, indicating that the subunits are, within the errors associated with a medium-resolution structure, essentially identical. We therefore detail only subunit A, unless stated otherwise.

### Overall structure

**Fig. 2:**
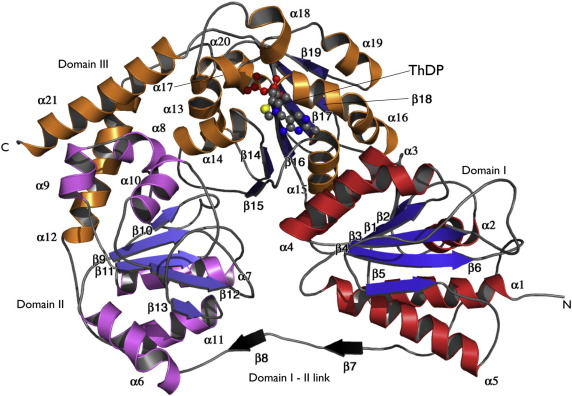
The secondary and domain structure of *Bs*MenD. A ribbon diagram of the enzyme subunit showing the elements of secondary structure with atoms of ThDP depicted as spheres according to atom type: C, gray; N, blue; O, red; S, yellow; P, orange. The N- and C-terminal positions are labeled, as are the elements of secondary structure.

MenD shows the three-domain architecture typical of ThDP-dependent enzymes (Fig. 2).^21^ Sequence and structural analyses place MenD in the pyruvate oxidase class of these enzymes, where domain I contains the cofactor pyrimidine-binding motif, domain III binds the diphosphate, and the central domain has variable, often ill-defined, function. In *Bs*MenD, domain I spans approximately the first 190 residues. An ordered linker region leads into domain II, which consists of residues 205–345. A smaller linker then leads into domain III, which starts at approximately residue 355 and continues to the C terminus (Fig. 2). Each domain consists of a parallel β-sheet sandwiched between several α-helices; in *Bs*MenD, the central domain has five strands and there are six in domains I and III.

### Quaternary structure

**Fig. 3:**
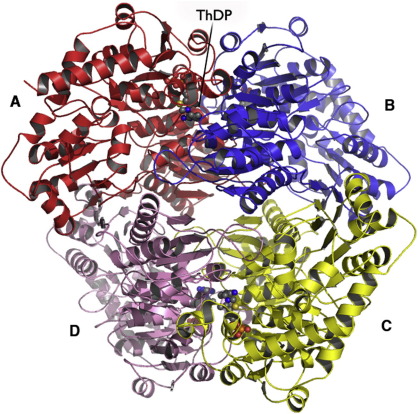
The *Bs*MenD tetramer. The tetramer is shown as a van der Waals surface with subunits labeled and colored differently. Subunit A is in the same orientation as in Fig. 2.

Oligomerization is essential for the function of ThDP-dependent enzymes, with each active site formed from two subunits.^21^ Most of these enzymes function as either dimers or tetramers. Size-exclusion chromatography indicated that *Bs*MenD consists of a mixture of dimer and tetramer in solution, with the dimer being much more abundant. The crystal structure of *Bs*MenD consists of a dimer-of-dimers (Fig. 3); a similar result was found for *Ec*MenD, which is predominantly dimeric in solution^15^ and then forms a dimer-of-dimers when crystallized.^15–17^

Analysis using the Protein Interfaces, Surfaces and Assemblies server^22^ suggests that the dimer-of-dimers is the most stable oligomeric form, burying approximately 23% (5310 Å^2^) of the surface area of each subunit (23,100 Å^2^). The principal interface, between subunits A and B (and C and D), buries about 14% of the surface area of each subunit. The A:B interface covers an area of about 3130 Å^2^, and the C:D interface covers approximately 3160 Å^2^. Around 30 hydrogen bonds in addition to the salt bridges formed between Arg53 and Asp55 with their counterparts in the second dimer form the most important interactions in this interface (data not shown). Fewer hydrogen bonds link the dimer-of-dimers arrangement where the pairs of subunits involved are A:D and B:C. This interface is formed primarily by residues located in the linker between domains I and II. A short three-amino-acid stretch of β-strand is present at the start of domain II, which forms an extension of the parallel β-sheet of domain II across the interface. In addition, another three-residue stretch (205–207) forms a two-stranded antiparallel β-sheet across the interface. Approximately 7% of the surface area of a subunit is buried at this interface; 1610 and 1620 Å^2^ for the A:D and B:C interface regions, respectively. The A:C and B:D interfacial regions occlude about 700 and 710 Å^2^, respectively, each representing approximately 2% of the subunit surface area.

### Comparison with *Ec*MenD

**Fig. 4:**
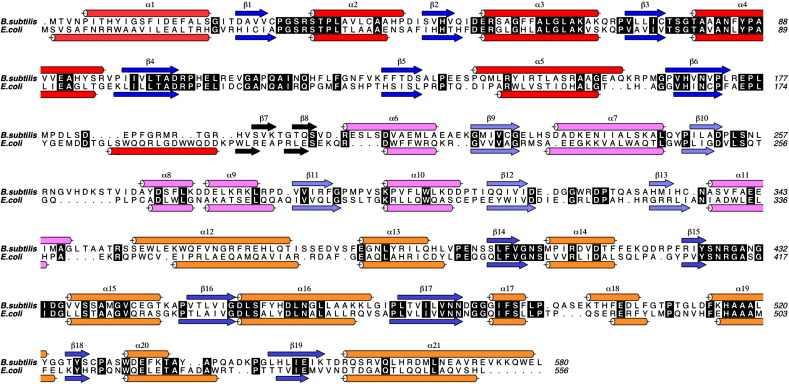
The primary and assigned secondary structure of *Bs*MenD and *Ec*MenD. α-Helices are shown as cylinders and β-strands as arrows, colored according to the domain to which they have been assigned (see Fig. 2). Residues with a black background are strictly conserved.

The MenD proteins from different organisms share little sequence identity, typically in the range 20–30%. Such a low level of sequence conservation despite the same function appears to be a common feature of enzymes that are dependent on ThDP.^15^ *Bs*MenD has 28% overall sequence identity to the *E. coli* protein. The sequence identity is higher for domains I and III (32% and 33%, respectively) compared to the central domain (16%). Nevertheless, the structures of the two enzymes are closely related, with the majority of secondary-structure elements conserved in each domain (Fig. 4). One exception is the final helix in domain I of *Ec*MenD, which is replaced by a loop and β7 in *Bs*MenD (Fig. 4). This region does not appear to have any functional role.

Subunit A from *Bs*MenD overlays with a subunit from *Ec*MenD [Protein Data Bank (PDB) code: 2jlc] with an r.m.s.d. of approximately 2.0 Å (over 503 aligned C^α^). The closeness of the structures extends to the dimer (r.m.s.d. of 2.0 Å over 1006 aligned C^α^) and the tetramer (2.4 Å over 1996 C^α^). Individual domains align more closely; domain I of *Bs*MenD overlays with an r.m.s.d. of 1.3 Å onto domain I of *Ec*MenD (173 aligned C^α^), domain II with an r.m.s.d. of 1.8 Å (117 aligned C^α^), and domain III with an r.m.s.d. of 1.7 Å (over 196 aligned C^α^). The loop containing residues responsible for binding to the diphosphate group of ThDP (approximately residues Gly486 to Pro509) is disordered in *Ec*MenD in the absence of cofactor^16^ but ordered in the holo-enzyme.^15^ The conformation of this cofactor-binding loop in holo-*Bs*MenD is similar to that of the corresponding loop in the holo-*Ec* structure (data not shown).

The low level of sequence identity between *Bs*MenD and *Ec*MenD serves to highlight the critical determinants of structure and function, and we placed a particular emphasis on conservation when analyzing potential contributions that residues make to enzyme activity. In the following text, unless stated otherwise, we confine our discussion to residues that are strictly conserved between *Bs*MenD and *Ec*MenD.

### Cofactor binding and the active site

**Fig. 5:**
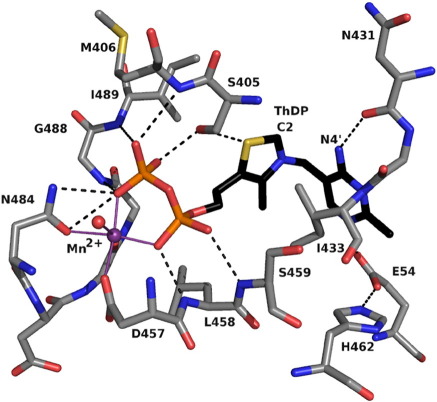
ThDP interactions with *Bs*MenD. Atoms are colored as follows: C of ThDP, black; C of protein, gray for subunits A and B; P, orange; N, blue; O, red; S, yellow; Mn^2+^, purple. Purple continuous lines represent coordination of the transition metal ion, and black broken black lines represent potential hydrogen-bonding interactions.

Dimerization is essential for formation of the cofactor binding and active site, with residues from domain I of subunit A and domain III from subunit B forming one site (and *vice versa*, leading to two active sites per dimer, Fig. 3). A metal cation, assigned as Mn^2+^, is bound and helps to position the diphosphate group of ThDP and so tether one part of the cofactor to the protein. This is a common feature of ThDP binding and similar to what is observed in *Ec*MenD.^15^ In *Bs*MenD, the side chains of Asp457 and Asn484, the main-chain carbonyl of Gly486, a water molecule, and two oxygen atoms from the diphosphate group form an octahedral coordination sphere about the metal ion (Fig. 5).

Almost all ThDP-dependent enzymes have a glutamate interacting with N1′ of the pyrimidine; Glu54 fulfills this role in *Bs*MenD. Mutagenesis of the corresponding residue, Glu55, in *Ec*MenD completely abolished enzyme activity.^7^ The main-chain amide of Ile433 and carbonyl of Asn431, which participate in hydrogen-bonding interactions with N3′ and N4′, respectively, are from the partner subunit. The side chain of Ile433, a residue strictly conserved in MenD sequences,^15^ is placed to interact with both the pyrimidine and thiazolium rings helping to hold the cofactor in a V shape, bent at C7′. All ThDP-dependent enzymes that have been structurally characterized have a large hydrophobic side chain in exactly this position. The environment of the cofactor forces N4′ on the pyrimidine ring into close contact with C2 in the thiazolium ring (3.1 Å, Fig. 5), activating the cofactor to contribute to catalysis.

ThDP binding involves contributions from a pair of subunits. The diphosphate is bound between the N-terminal section of α14, strands β16 and β17, and the loop between β17 and α17. The pyrimidine moiety is positioned near the loop linking β15–α15. These are all from one subunit. The partner subunit contributes to binding the pyrimidine with contributions from the C-terminal sections of β1 and β3, the loop between β3 and α4, together with an important contribution from Glu54 at the N terminus of α3. The thiazolium group is sandwiched between Ile433 (on the loop linking β15 with α15) and Phe490 (α17).

**Fig. 6:**
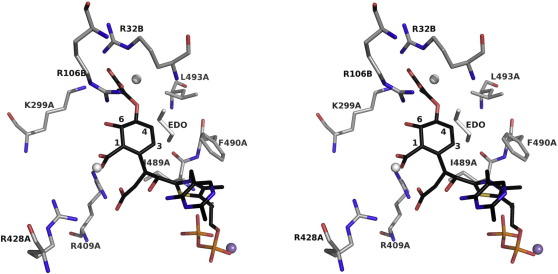
A model for the post-decarboxylation covalent adduct formed after ThDP C2 has reacted with α-ketoglutarate and then isochorismate. A stereoview into the active site with atoms colored as follows: C of ThDP, black; C of protein, gray; O, red; N, blue; P, orange; Mn^2+^, purple. The eight key residues discussed in the text are labeled. Two water molecules are depicted as gray spheres and ethane-1,2-diol is shown as gray sticks, labeled EDO.

The narrow crevice where substrates bind is likewise formed by a dimer. One subunit contributes three α-helical segments (α10, α14, and α17), which line one side of the cavity. Three non-helical segments (the β1–α2 turn, the loop between β6 and β7, and the loop following β4) are provided by the partner subunit. The active site is polar, primarily basic due to the presence of four arginine residues (32A, 106A, 409B, and 428B) and a lysine (299B). A hydrophobic patch is formed by Ile489B, Phe490B, and Leu493B (Fig. 6). Seven of these eight amino acids are strictly conserved, and one, Lys299, is conserved or replaced by arginine in more than 90% of MenD sequences.

Ser405 OG donates a hydrogen bond to one of the phosphate groups of ThDP yet is placed about 3.1 Å from the thiazolium S. This alignment of functional groups may induce a polarization effect on the S atom and contribute to generation of an ylide.

### A proposed mechanism

**Fig. 7:**
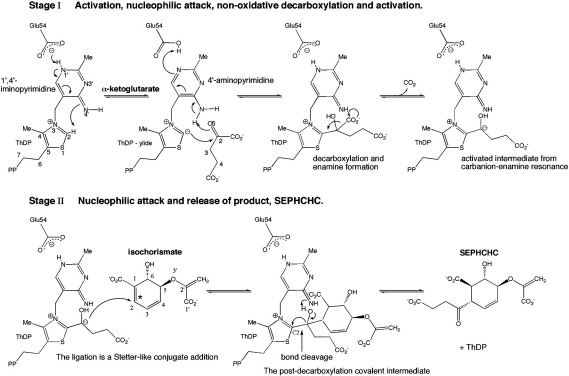
A two-stage mechanism for catalysis by *Bs*MenD. An asterisk (⁎) marks the isochorismate C2, which is attacked by the carbanion intermediate. The post-decarboxylation covalent intermediate in stage II is the structure that has been modeled and is shown in Fig. 6.

The structure of MenD and our model (Fig. 6) of the activated intermediate–isochorismate complex suggested that the actual enzyme mechanism is driven by the chemical properties of the cofactor ThDP. A two-stage mechanism that corresponds to reactions with each of the substrates can be proposed (Fig. 7).^15^ The absence of any residue that can act as a general acid/base indicates that the cofactor N4′ is a critical component of the mechanism. Such an observation is consistent with structural and mechanistic studies of another ThDP-dependent enzyme, *N*^2^-(2-carboxyethyl)arginine synthase.^23^

Our model for activity predicts that five polar residues interact with carboxylates and hydroxyl group of substrates to align reagents for catalysis and that non-polar components of isochorismate interact with the hydrophobic patch. The structural studies, sequence comparisons, molecular modeling, and mechanistic considerations lead us to conclude that the environment provided by MenD for the cofactor and the chemical properties of ThDP provide the functionality for catalysis. A first approximation of how substrates bind can then be used to address the molecular recognition of these substrates.

### Mutagenesis and kinetic studies

Attempts to soak ligands such as α-ketoglutarate and salicylic acid into the crystals of *Bs*MenD and thus derive structural data directly relevant to substrate recognition resulted in the loss of diffraction. Co-crystallization experiments were also carried out but either no crystals were observed or, where analyzed, there was no electron density that could be attributed to these ligands (data not shown). A model of the post-decarboxylation covalent adduct formed after reaction of ThDP with first α-ketoglutarate then isochorismate (Figs. 6 and 7) was constructed, guided by the principle that important features relevant to substrate recognition and catalysis should be conserved between *Bs*MenD and *Ec*MenD. We also noted that the positions of two water molecules and ethane-1,2-diol in the active sites serve to identify potential ligand interacting sites and provide a hint of where functional groups of isochorismate might be placed (Fig. 6).

Modeling of substrate binding, sequence, and structure comparisons identified eight residues that are potentially important with respect to substrate recognition. The first stage of the reaction involves the formation of a covalent adduct between the cofactor and α-ketoglutarate; the formation of such species is well established in ThDP-dependent enzymes^24^ and the adduct structure has been captured crystallographically.^25^ The driving force for this stage largely comes from the geometry of the cofactor in the binding site, which brings N4′ of the pyrimidine ring and C2 of the thiazolium ring into close proximity. N4′ abstracts the acidic proton from C2, generating a carbanion ylide, which can then attack the first substrate, α-ketoglutarate, to form the first covalent intermediate (Fig. 7). Arg409 and Arg428 (Fig. 5) are well placed to interact with the α-ketoglutaric acid part of this adduct. Mutation of these residues to alanine had a significant effect on the binding of α-ketoglutarate and the rate of reaction (Table 2). The Arg409Ala mutation resulted in a 7-fold increase in *K*_m_ and 5-fold decrease in *k*_cat_. The Arg428Ala mutation gave an increase in *K*_m_ of more than 20-fold and a 4-fold decrease in *k*_cat_. These mutations resulted in comparable *k*_cat_/*K*_m_ values, which are significantly decreased, compared to the wild-type enzyme. Assay of these two mutants with respect to isochorismate indicates that Arg409 has a greater influence on the second substrate binding since the *K*_m_ is increased 8-fold with no change to *K*_m_ of the Arg428Ala mutant (Table 3). Although catalytic efficiency is compromised in both cases, ascribed to poorer binding of α-ketoglutarate in the first stage of the reaction, the Arg409Ala mutant is affected to a much larger degree with a reduction in *k*_cat_/*K*_m_ by 2 orders of magnitude. These data suggest then that Arg409 is important for binding of both substrates whereas Arg428 primarily contributes to the binding of α-ketoglutarate. This is consistent with the crystal structure since the side chain of Arg409 is about 1 Å closer to the thiazolium group than Arg428, at the base of the active site cleft directed towards the cavity where the second substrate binds.

Arg32 and Arg106 are in close proximity on one side of the active-site entrance; Lys299 is about 8 Å distant on the other side of the cleft. Arg106 is the closest to the thiazolium group at a distance of about 9 Å. The model of the covalent or activated intermediate suggests that this triumvirate of basic residues is likely to have a small influence, if any, on α-ketoglutarate binding. The kinetic data (Table 2) indeed suggest that this is the case. There is effectively no change to *K*_m_ with respect to α-ketoglutarate for the Arg32Ala and Lys299Ala mutants, and *K*_m_ is reduced by a factor of 3 for the Arg106Ala protein. With respect to isochorismate (Table 3), the Lys299Ala mutant displays an increase in *K*_m_ of about 8-fold but with a comparable *k*_cat_ value, whereas the mutations to the two arginine residues increase *K*_m_ between 40- and 50-fold and reduce *k*_cat_ significantly. The reduction in catalytic efficiency is most pronounced for the Arg106 mutation. These data are consistent with Arg106 performing an important role in recognition and binding of the second substrate.

Three hydrophobic residues, Ile489, Phe490, and Leu493, cluster on one side of the active site (Fig. 6). Of these, Leu493 is most distant, about 9.5 Å from the thiazolium. The kinetic data indicate a minor perturbation of *Bs*MenD activity when Leu493 is mutated to alanine and this is mainly due to an increase in *K*_m_ for isochorismate. Leu493 does not therefore appear to make a particularly significant or direct contribution to reactivity. The changes to kinetic properties may be due to perturbation of the hydrophobic cluster since the other two residues appear more important. Mutation of Ile489 and Phe490 increases the *K*_m_ for α-ketoglutarate 16-fold and 12-fold, respectively. The *K*_m_ for isochorismate is increased by 24- and 25-fold, respectively, and there is a concomitant reduction in catalytic efficiency. Ile489 is adjacent to the site where α-ketoglutarate reacts with the thiazolium group, 3.8 Å distant from the sulfur atom. Phe490 participates in van der Waals interactions with the thiazolium and Ile489. These two residues appear therefore to help bind ThDP. Ile489 is likely to interact with the aliphatic part of the intermediate formed after α-ketoglutarate has reacted with ThDP and, together with Phe490, to interact with the hydrophobic component of the isochorismate ring, C3 and C4 (Fig. 6).

In the second stage of the reaction, the carbanion adduct attacks isochorismate C2 (Fig. 7). Two further arginine residues (Arg32 and Arg106) and a lysine (Lys299) line one side of the putative isochorismate-binding region. A hydrophobic patch consisting of Ile489, Phe490, and Leu493 (Fig. 6) lines the opposite side of the active site. Kinetic characterization of Ala mutants of each of these residues is summarized in Tables 2 and 3, with respect to both substrates. Mutation has little effect on the binding of ThDP (data not shown).

Lys299, the only highlighted residue located in the central domain, appears to have little influence on the reaction. Mutation of any of the arginine residues has a similar effect on the binding of isochorismate; however, alteration to Arg106 has a greater impact upon the rate of reaction. This residue is located at the start of the long loop linking β4 with β5; in addition to its putative role in the reaction, Arg106 is well placed to form a hydrogen bond with the main-chain carbonyl of Pro116, helping to orient this loop, which forms part of the binding pocket. Removal of this interaction could destabilize the loop. Of the hydrophobic residues, mutation of Phe490 has the most deleterious effect, with respect to both substrates, indicating particular importance in defining the overall shape and size of the binding pocket.

### Concluding remarks

This combination of single crystal diffraction methods with site-directed mutagenesis and kinetic analyses has allowed us to elucidate the structure–reactivity relationship that governs MenD activity. The enzyme provides the environment to bind the cofactor in a specific manner such that the mechanism is driven by the chemical properties of ThDP. There is no requirement for any amino acid to contribute directly to the mechanism by abstraction or provision of a proton. Rather, the residues in the active site are important for the binding and orientation of substrates allowing MenD to accomplish catalysis. The mutagenesis data are consistent with such a conclusion since they indicate that the most profound effect on catalytic efficiency is derived from alteration of non-polar residues implicated in substrate recognition.

## Materials and Methods

### Reagents, preparation of recombinant expression systems, and protein purification

Chemicals and materials were sourced from Sigma-Aldrich and VWR International except where stated otherwise. The *menD* gene was amplified by PCR from *B. subtilis* genomic DNA (ATCC strain 23857, LGC Standards Office, UK) with 5′-**CTCGAG**ATGACAGTGAACCCAATTAC-3′ and 5′-**GGATCC**TTACAGTTCCCATTGTTTTTTC-3′ as the forward and reverse primers, respectively (Thermo Scientific). These oligonucleotides include 5′ XhoI and 3′ BamHI restriction sites, respectively, indicated in boldface. The PCR product was ligated into TOPO-BLUNT-II (Invitrogen) and then sub-cloned into a modified pET15b vector (Novagen), which produces a histidine-tagged protein with a tobacco etch virus protease site. The hexa-His tag, tobacco etch virus cleavage site, and additional residues from the choice of cloning sites added a total of 24 extra amino acids at the protein N terminus. The native and SeMet-labeled proteins were obtained using *E. coli* strains BL21(DE3) and the methionine auxotroph B834 (DE3) (Stratagene), respectively. Cells were grown in 1 L of Luria–Bertani media supplemented with 50 μg mL^− 1^ of carbenicillin for production of native protein, while for the SeMet protein, bacteria were cultured in minimal media (Molecular Dimensions) supplemented with SeMet following an established protocol.^26^ Gene expression was induced at 20 °C using 0.5 mM isopropyl-β-d-thiogalactopyranoside, and growth continued for 16 h at room temperature.

The same purification protocol was used for both native and SeMet *Bs*MenD. Cells were harvested by centrifugation for 25 min at 40,000***g*** at 4 °C, re-suspended in lysis buffer (50 mM Tris–HCl, pH 7.5, 250 mM NaCl, and 20 mM imidazole) containing DNase I (0.1 mg) and a single tablet of a cocktail of ethylenediaminetetraacetic acid-free protease inhibitors (Roche), and lysed using a French press at 1000 psi. Insoluble debris was separated by centrifugation at 39,000***g*** for 25 min at 4 °C, and the soluble fraction was loaded onto a 5-mL HisTrap HP column (GE Healthcare) pre-charged with Ni^2+^. A linear concentration gradient was applied to the column and *Bs*MenD eluted at a concentration of 250 mM imidazole. Fractions were analyzed using SDS-PAGE, and those containing *Bs*MenD were pooled. The protein, still carrying the affinity tag, was further purified using a Superdex 200 26/60 size-exclusion column (GE Healthcare) equilibrated with 50 mM Tris–HCl and 250 mM NaCl, pH 7.5. This column had previously been calibrated with molecular mass standards, blue dextran (> 2000 kDa), thyroglobulin (669 kDa), ferritin (440 kDa), aldolase (158 kDa), conalbumin (75 kDa), ovalbumin (43 kDa), carbonic anhydrase (29.5 kDa), ribonuclease A (13.7 kDa), and aprotinin (6.5 kDa) (GE Healthcare; data not shown). Selected fractions were pooled and dialyzed into 20 mM Tris–HCl and 50 mM NaCl, pH 7.5, and concentrated using Amicon Ultra devices (Millipore) to 8 mg mL^− 1^ for the native protein and 4 mg mL^− 1^ for the SeMet sample. The protein concentration was determined spectrophotometrically using a theoretical extinction coefficient of 52,745 M^− 1^ cm^− 1^ at 280 nm calculated using ProtParam.^27^ The full incorporation of SeMet and high level of sample purity for both samples was verified by matrix-assisted laser desorption/ionization time-of-flight mass spectrometry and SDS-PAGE (data not shown). The native protein expressed at levels in excess of 150 mg L^− 1^ of culture; yields of the SeMet protein were lower, typically about 60 mg L^− 1^, due to the lower bacterial cell mass obtained in minimal media.

The native protein typically eluted from the gel-filtration column in two peaks: a major peak with a mass of approximately 150 kDa and a minor peak of with a mass of approximately 290 kDa. The theoretical mass of a *Bs*MenD subunit is approximately 64.1 kDa, and therefore, the peaks correspond to a dimer and tetramer, respectively. Approximately 10% of the total protein was observed in the minor peak, which was discarded, and the major peak was used subsequently.

### Crystal growth, structure determination, and refinement

#### Crystallization and data collection

Sitting and hanging drop vapor diffusion methods were used to screen and optimize crystallization conditions, respectively, based on commercially available screening sets. The *Bs*MenD:ThDP:Mn^2+^ complex crystallized with the N-terminal hexa-His tag intact whereas the apo-enzyme did not crystallize at all. Holo-enzyme was prepared by incubation with 2 mM MnCl_2_ and 0.5 mM ThDP, and crystals were obtained using a reservoir (500 μL) comprising 35% polyethylene glycol (PEG) 1.5K, 0.1 M Tris, pH 8, and 0.25 M (NH_4_)_2_SO_4_, for the native protein and 24% PEG 3K, 0.1 M Tris, pH 8, and 0.2 M Li_2_SO_4_ for SeMet *Bs*MenD. In both cases, the best crystals were obtained from drops consisting of 1 μL protein and 2 μL of reservoir. Crystals appeared as rectangular prisms, consistently with a notch at one end. The maximum dimensions of native crystals were 0.3 mm × 0.1 mm × 0.1 mm, and the SeMet derivative crystals were smaller at 0.2 mm × 0.1 mm × 0.1 mm.

Crystals were flash cooled in liquid nitrogen, mounted on a goniostat, and maintained at − 173 °C in a flow of cooled nitrogen, and diffraction properties were characterized with a Rigaku Micromax 007 rotating anode R-AXIS IV^++^ image plate system. The best samples were stored for use at the European Synchrotron Radiation Facility (Grenoble, France). Crystals of both proteins, particularly the SeMet crystals, were radiation sensitive. Native data were collected on beam line ID23-1, and SAD data were measured on beam line BM14, both with ADSC Q315 CCD detectors. The SAD data were collected at the Se K-absorption edge, determined using X-ray Absorption Near Edge Structure spectroscopy to provide the *f*″ maximum at a wavelength of 0.97864 Å. Data were integrated using XDS^28^ and scaled with XSCALE (SeMet)^28^ or SCALA (native),^29^ and statistics are presented in Table 1.

#### Structure solution and refinement

Attempts to solve the *Bs*MenD structure by molecular replacement using the *Ec*MenD mode (PDB code: 2jlc) gave ambiguous results; the sequence identity shared between the two is 28%. Since the asymmetric unit was predicted to contain 68 methionine residues, based on four subunits per asymmetric unit, it was judged likely that a SeMet-SAD experiment would provide useful phase information.

Analysis of the SAD data using XPREP^30^ indicated that the anomalous dispersion signal/noise ratio reduced to a value of 1.0 at 3 Å resolution and these data were input to the program SOLVE.^31^ Initial phases were obtained by SAD phasing based on the positions of 30 selenium atoms. The initial figure of merit was 0.25 with a *Z*-score of 62.3. Density modification using RESOLVE^32^ with an estimated solvent content of 55% improved the figure of merit to 0.75. Automated map interpretation with RESOLVE produced a partial model consisting of approximately 1300 residues split over 52 polypeptide chains. This model was extended using Coot^33^ with the benefit of the *Ec*MenD structure as a guide. The major secondary-structure elements of a single subunit were completed, and the resulting structure was used as a molecular replacement model for the native data, using Phaser.^34^ The four subunits present in the asymmetric unit were all located with *Z*-scores above 12, indicating correct solutions. Further manual rebuilding, the placement of the cofactor ThDP, Mn^2+^ and SO_4_^2^^−^ ions and waters using Coot, and incorporation of dual rotamer side chains interspersed with refinement using REFMAC5.^35^ A strong electron density feature was observed in each active site and this was successfully modeled and refined as ethane-1,2-diol, a precursor of and potential contaminant in PEGs.^36^ Strict non-crystallographic symmetry restraints were applied during the initial stages of refinement; these were relaxed and then removed as the refinement progressed. Model geometry statistics are presented in Table 1. Subunits A and D are involved in more contacts with symmetry-related molecules, leading to significantly lower B-factors for these chains (Table 1). Figures were prepared with Chemdraw (Adept Scientific), PyMOL,^37^ and ALINE.^38^

### Mutagenesis and kinetic studies

The QuikChange Site-Directed Mutagenesis Kit (Stratagene) was used to introduce eight point mutations into *Bs*MenD. The primers used to generate the mutants are listed in Supplemental Table 1. Expression and purification of the mutants followed the procedures described above for the native protein. Preparation and purification of isochorismate followed previous protocols.^39^ Protein activity was measured by monitoring the decrease in absorption at 278 nm due to the consumption of isochorismate.^8^ All kinetic assays were carried out in 50 mM Tris–HCl, pH 7.8, with 20 mM NaCl and 5 mM MgSO_4_ at 23 °C. Circular dichroism measurements indicated that in each case, the mutated protein was still correctly folded (data not shown).

### PDB accession numbers

Coordinates and structure factors have been deposited with accession code 2x7j.

## Figures and Tables

**Table 1:** Crystallographic and model geometry statistics for *Bs*MenD

	SeMet derivative	Native
Resolution range (Å)	50–3.0^a^	68.8–2.35
Unit cell dimensions: *a*, *b*, *c* (Å)	100.1, 152.6, 158.1	100.2, 153.0, 158.5
Space group	*P*2_1_2_1_2_1_	*P*2_1_2_1_2_1_
Total/unique reflections	633,457/93,394	720,895/101,958
Redundancy/completeness (%)^b^	6.8^c^/99.7 (99.4)	7.1/100 (100)
Wilson *B* (Å^2^)	23.5	41.0
〈*I*/σ(*I*)〉	16.5 (7.1)	17.3 (4.1)
*R*_merge_	10.4 (27.5)	10.9 (39.4)
Protein residues (A/B/C/D)		579/579/577/579
Water molecules/ThDP/Mn^2+^		1075/4/4
SO_4_^2−^/ethane-1,2-diol		8/4
*R*_work_/*R*_free_/DPI (Å)^d^		17.3/22.9/0.23
Average *B*-factors (Å^2^)		
Overall/side chain/main chain		
Subunit A		31.3/31.8/30.8
Subunit B		35.9/36.3/35.5
Subunit C		35.3/35.7/34.9
Subunit D		30.4/30.8/30.1
Water molecules/ThDP/Mn^2+^		34.7/23.0/25.8
SO_4_^2−^SO_4_^2-^/ethane-1,2-diol		20.2/28.4
r.m.s.d. bond lengths (Å)/angles (°)		0.009/1.32
Ramachandran plot analysis (%)^e^		
Favorable/outliers		96.8/0.6

a The limit of the useful anomalous signal.

b Values in parentheses refer to the highest-resolution shell; 3.16–3.0 Å (SeMet), 2.41–2.35 Å (native).

c Friedel pairs treated separately.

d The diffraction-component precision index as defined by Cruickshank.^18^

e According to MolProbity.^19^

**Table 2:** Steady-state kinetic parameters with respect to α-ketoglutarate

Protein	*K*_m_ (μM)	*k*_cat_ (min^− 1^)	*k*_cat_/*K*_m_ (μM^− 1^ min^− 1^)	Relative *k*_cat_/*K*_m_
Wild type	22 ± 5	14 ± 2	0.64 ± 0.3	1
R409A	159 ± 18	2.7 ± 0.3	0.16 ± 0.02	0.03
R428A	514 ± 54	3.4 ± 0.2	0.007 ± 0.001	0.01
R32A′	22 ± 4	4.8 ± 0.4	0.22 ± 0.02	0.3
R106A′	7.7 ± 2.2	0.6 ± 0.2	0.09 ± 0.03	0.1
K299A	22 ± 4	22 ± 2	1 ± 0.2	1.5
L493A	18 ± 1	10 ± 1	0.5 ± 0.1	0.8
I489A	346 ± 10	5.7 ± 0.2	3.1 ± 0.1	0.01
F490A	274 ± 12	1.1 ± 0.1	0.0039 ± 0.0002	0.006

Residues marked with ′ are located in the second subunit forming the active site.

**Table 3:** Steady-state kinetic parameters with respect to isochorismate

Protein	*K*_m_ (μM)	*k*_cat_ (min^− 1^)	*k*_cat_/*K*_m_ (μM^− 1^ min^− 1^)	Relative *k*_cat_/*K*_m_
Wild type	1.1 ± 0.2	19 ± 1	19 ± 3	1
R409A	8.5 ± 1.2	1.4 ± 0.1	0.16 ± 0.02	0.009
R428A	1.1 ± 0.5	2.8 ± 0.8	2.8 ± 0.8	0.15
R32A′	49 ± 7	11 ± 1	0.22 ± 0.01	0.01
R106A′	41 ± 4	0.7 ± 0.2	0.018 ± 0.01	0.00095
K299A	8.4 ± 1.2	25 ± 2	3.0 ± 0.3	0.16
L493A	39 ± 1	19 ± 1	0.49 ± 0.03	0.03
I489A	27 ± 1	5.7 ± 0.2	0.21 ± 0.01	0.01
F490	29 ± 3	1.7 ± 0.1	0.058 ± 0.01	0.003

Residues marked with ′ are located in the second subunit forming the active site.

## Appendix A

Supplemental Table 1 Oligodeoxynucleotide primers used in site-directed mutagenesis modification of *Bs*MenD.
