# Supplementary material for: Structure and Reactivity of Bacillus subtilis MenD Catalyzing the First Committed Step in Menaquinone Biosynthesis
Source: J Mol Biol. 2010 Aug 13;401(2):253–64. doi: 10.1016/j.jmb.2010.06.025 (PMC2914249; doi:10.1016/j.jmb.2010.06.025)
Supplement: Supplemental Table 1 — Oligodeoxynucleotide primers used in site-directed mutagenesis modification of BsMenD. [file mmc1.doc]

Supplemental Table 1. Oligodeoxynucleotide primers used in site-directed mutagenesis modification of *Bs*MenD.

| **Mutant** | **Primer** | **Primer sequence** |
| --- | --- | --- |
| R32A | forward  reverse | gtttgtccgggctcagcatctacaccgctggct  agccagcggtgtagatgctgagcccggacaaac |
| R106A | forward  reverse | gtgttaactgctgatgcgcctcatgagctgcgc  gcgcagctcatgaggcgcatcagcagttaacac |
| K299A | forward  reverse | ccgatgcctgtttcagcaccggttttcttatgg  ccataagaaaaccggtgctgaaacaggcatcgg |
| R409A | forward  reverse | aacagcatgccgatcgcggatgttgatacgttt  aaacgtatcaacatccgcgatcggcatgctgtt |
| R428A | forward  reverse | cggatttattcaaacgcgggagcaaacgggata  tatcccgtttgctcccgcgtttgaataaatccg |
| I489A | forward  reverse | aatgacggaggaggggctttctcctttttgccg cggcaaaaaggagaaagcccctcctccgtcatt |
| F490A | forward  reverse | gacggaggagggattgcctcctttttgccgcag ctgcggcaaaaaggaggcaatccctcctccgtc |
| L493A | forward  reverse | gggattttctcctttgcgccgcaggcttctgag ctcagaagcctgcggcgcaaaggagaaaatccc |
